# An Account of the Fox Glove, and Some of Its Medical Uses; with Practical Remarks on Dropsy, and Other Diseases

**Published:** 1785

**Authors:** 


### BOOKS.

I. *An Account of the Fox Glove, and some of its Medical Uses; with practical Remarks on Dropsy, and other Diseases. By* William Withering, *M. D. Physician to the General Hospital at Birmingham.* 8vo. Birmingham, 1785. 207 pages *.

IN his introduction to this valuable work Dr. Withering acknowledges himself indebted to empirical practice for his first knowledge of this remedy, which, it seems, he has employed since the year 1775 in one hundred and sixty-three cases, the results of which, whether successful or unsuccessful, are candidly related in the work before us, together with many other cases communicated by correspondents.

From the whole of these cases Dr. Withering infers, 1. that although the digitalis pur-

* Prefixed to the work is a coloured figure of the plant, copied, by permission of Mr. Curtis, from his excellent work, the *Flora Londinensis*.

purea purea will not univerſally act as a diuretic, yet that 2. it will do ſo more generally than any other medicine; 3. that it will often produce this effect after every other probable method has been fruitleſſly tried; 4. that if this fails, there is but little chance of any other medicine ſucceeding; 5. that, in proper doſes, it is mild in its operation, and gives leſs diſturbance to the ſyſtem than ſquill, or almoſt any other active medicine; 6. that when dropſy is attended by palſy, unſound viſcera, great debility, or other complication of diſeaſe, neither the digitalis nor any other diuretic can do more than obtain a truce to the urgency of the ſymptoms, unleſs, by gaining time, it may afford opportunity for other medicines to combat and ſubdue the original diſeaſe; 7. that the digitalis may be uſed with advantage in every ſpecies of dropſy, except the encyſted; 8. that it may be made ſubſervient to the cure of diſeaſes unconnected with dropſy; and 9. that it has a power over the motion of the heart to a degree yet unobſerved in any other medicine, and that this power may be converted to ſalutary ends. This particular effect of the digitalis, which we have frequently had occaſion to obſerve in our own trials with this medicine, is indeed very remarkable. Dr. Withering has ſeen the pulſe reduced by it even to thirty-five ſtrokes in a minute; and although this is the more uſual effect of large and quickly-repeated doſes of the medicine, yet caſes have occurred to him in which the pulſe has been retarded to an alarming degree, without any other preceding effect.

In treating of the preparations and doſes of the digitalis, Dr. Withering gives the preference to the leaves. Theſe he adviſes to be gathered after the flowering ſtem has ſhot up, and about the time that the bloſſoms are coming forth. The leaf ſtalk and mid-rib of the leaves are to be rejected, and the remaining part dried, either in the ſunſhine, or on a tin pan or pewter diſh before a fire.

If well dried, he obſerves, they may eaſily be reduced to a beautiful green powder, which weighs ſomething leſs than one fifth of the original weight of the leaves.

To adults our author gives from one to three grains of this powder twice a day. In the reduced ſtate in which phyſicians generally find dropſical patients, he thinks four grains a day will be ſufficient. He ſometimes gives the powder alone; ſometimes unites it with aromatics; matics; and ſometimes forms it into pills with a quantity of ſoap or gum ammoniac.

If a liquid medicine be preferred, he orders a drachm of theſe dried leaves to be infuſed for four hours in half a pint of boiling water, adding to the ſtrained liquor an ounce of any ſpirituous water. One ounce of the infuſion given twice a day is a medium doſe for an adult patient. If the patient be ſtronger than uſual, or the ſymptoms very urgent, this doſe, the author obſerves, may be given once in eight hours; and, on the contrary, in many inſtances, he adds, half an ounce at a time will be ſufficient. About thirty grains of the powder, or eight ounces of the infuſion, may generally, we are told, be taken before the nauſea commences.

We formerly * had occaſion to remark, that the diuretic effect of this medicine ſeemed to be independent of the ſickneſs it excites; farther experience has ſince abundantly convinced us of the truth of this obſervation, and in the work now before us, Dr. Withering informs us that he ſoon learnt that the diuretic effects of the digitalis would often take place firſt, and

ſometimes fometimes be checked when ficknefs or purging fupervened. This obfervation, added to the attention required to the ftate of the pulfe, has long convinced him that it is of confequence not to repeat the dofes too quickly, but to allow fufficient time for the effects of each to take place, as he has found it very poffible to pour in an injurious quantity of the medicine before any of the fignals for forbearance have appeared. "Let the medicine "therefore," fays he, "be given in the dofes "and at the intervals mentioned above; let it "be continued until it acts on the kidneys, "the ftomach, the pulfe, or the bowels; let "it be ftopped upon the firft appearance of "any one of thefe effects, and I will maintain "that the patient will not fuffer from its exhi-"bition, nor the practitioner be difappointed "in any reafonable expectation."

Dr. Withering advifes a liberal ufe of diluents during the operation of the medicine. In cafes of afcites and anafarca, when the patients are weak, and the evacuation of the water rapid, the ufe of proper bandages, we are told, is indifpenfably neceffary to their fafety.

If the water fhould not be wholly evacuated, he thinks it right to allow an interval of feveral days days before the medicine be repeated, that food and tonics may be adminiftered. Of the ufual tonic medicines, however, he obferves, that in thefe cafes they have very often deceived his expectations.

From fome cafes which have occurred to him lately, he is difpofed to believe that the digitalis may be given in fmall dofes of two or three grains a day, fo as gradually to remove a dropfy without any other than mild diuretic effects, and without any interruption to its ufe until the cure be completed.

With regard to the circumftances of conftitution, which are more or lefs favourable to the fuccefs of the digitalis, Dr. Withering obferves that it feldom fucceeds in men of great natural ftrength, of tenfe fibres, of warm fkin, of florid complexion, or in thofe with a tight and cordy pulfe; that if the belly in afcites be tenfe, hard, and circumfcribed, or the limbs in anafarca folid and refifting, we have but little to hope; but that, on the contrary, if the pulfe be feeble or intermitting, the countenance pale, the lips livid, the fkin cold, the fwollen belly foft and fluctuating, or the anafarcous limbs readily pitting under the preffure of of the finger, we may expect the diuretic effects to follow in a kindly manner.

In cases which foil every attempt at relief, he has been aiming, for some time past, to make such a change in the constitution of the patient as might give a chance of success to the digitalis. By blood-letting, by neutral salts, chrystals of tartar, squills, and occasional purging, he has succeeded, though imperfectly. Next to the use of the lancet, he thinks nothing lowers the tone of the system more effectually than the squill, and consequently that it will always be proper, in such cases, to use the squill, because if that fails in its desired effect, it is one of the best preparatives to the adoption of the digitalis.

In a copy of Parkinson's Herbal our author has found a manuscript note written, he believes, by a Mr. Saunders, formerly an apothecary at Stourbridge, in which the digitalis is recommended as an infallible remedy in consumptions. This induces him to wish it a farther trial in this disease, although in his hands, he observes, it has done but little service.

In speaking of the phthisis pulmonalis, Dr. Withering mentions an unusual dilatation of the the pupil of the eye as the moſt certain characteriſtic of the diſeaſe.

In the work itſelf, to which we muſt now refer the reader, will be found many other valuable practical obſervations; but we cannot conclude the preſent account of it without expreſſing our approbation of the great prudence and caution the author uniformly inculcates in the uſe of this new remedy. That his ſolicitude to ſee it adminiſtered in ſmall doſes, and according to the rules he has laid down, is well founded, we are convinced, from two inſtances of its virulent effects, when given in large doſes, which have lately come to our knowledge.

